# Magnetically guided central nervous system delivery and toxicity evaluation of magneto-electric nanocarriers

**DOI:** 10.1038/srep25309

**Published:** 2016-05-04

**Authors:** Ajeet Kaushik, Rahul D. Jayant, Roozbeh Nikkhah-Moshaie, Vinay Bhardwaj, Upal Roy, Zaohua Huang, Ariel Ruiz, Adriana Yndart, Venkata Atluri, Nazira El-Hage, Kamel Khalili, Madhavan Nair

**Affiliations:** 1 Center for Personalized Nanomedicine, Institute of Neuroimmune Pharmacology, Department of Immunology, Herbert Wertheim College of Medicine, Miami, FL-33199, USA; 2 Department of Mechanical & Materials Engineering, College of Engineering and Computing, Florida International University, Miami, FL-33174, USA; 3 Department of Chemistry & Physics, Western Carolina University, Cullowhee - NC-28723, USA; 4 Department of Neuroscience, Center for Neurovirology and Comprehensive NeuroAIDS Center, Lewis Katz School of Medicine, Temple University, Philadelphia, PA-19140, USA

## Abstract

Least component-based delivery of drug-tagged-nanocarriers across blood-brain-barriers (BBB) will allow site-specific and on-demand release of therapeutics to prevent CNS diseases. We developed a non-invasive magnetically guided delivery of magneto-electric nanocarriers (MENCs), ~20 nm, 10 mg/kg, across BBB in C57Bl/J mice. Delivered MENCs were uniformly distributed inside the brain, and were non-toxic to brain and other major organs, such as kidney, lung, liver, and spleen, and did not affect hepatic, kidney and neurobehavioral functioning.

The brain can serve as a potential target for many deadly diseases, including tumors, neuro-AIDS and other neurodegenerative disorders^1^,^2^,^3^. However, most therapeutic drugs demonstrate only modest or no therapeutic benefits because these drugs are unable to pass through the blood brain barrier (BBB)^4^. The BBB has evolved to tightly control brain homeostasis by the presence of tight junctions between endothelial cells, feet of astrocytes as well as pericytes, which makes it impossible for the drugs to pass through^1^,^2^,^3^,^4^,^5^,^6^. Thus, investigating novel methodologies to deliver specific drugs across the BBB to treat CNS diseases is of great therapeutic significance^7^,^8^. Moreover, the investigation of new imaging tools for diagnosis, disease monitoring and treatment efficacies is receiving interest^9^. Nano-formulations (NFs), an ideal combination of nanomaterial i.e., nanocarrier and drugs display significant therapeutic efficacy against target diseases due to site-specific drug delivery and various stimulus-responsive release^10^,^11^,^12^,^13^,^14^. Unfortunately, most of the investigated NFs are only tested *in-vitro* or peripherally, and shows limited delivery across the BBB. Targeted drug delivery using special cells such as monocytes and macrophages, has also been investigated, but they too fail in efficiently delivering therapeutics across the BBB^14^,^15^,^16^. Another limitation of these methods is poor efficacy, as the delivery methods are not controlled, carriers become entrapped in endosomal pathway and fails to carry drug to the target site, or the drug is exocytosed. Many NFs are >100 nm in size, which is much larger than the size of synaptic cleft. Therefore, they get filtered out by the reticuloendothelial system. Due to the size constraints, 99% of NFs are deposited at major organs such as liver, lungs and lymphoid organs prior to their navigation across the BBB^1^,^2^,^3^,^17^.

Recently, ultrasound, alternate magnetic field, electric field, and photosensitization approaches have been introduced to increase the delivery and efficacy of NFs across the BBB^7^,^8^,^9^,^10^,^11^. However, these approaches are not well adopted as they offer transient BBB openings and exhibit side effects such as CNS cell damage and delayed recovery. Increased delivery of nanostructures of polymers and metals, including metal oxides and quantum dots, mediated by Trans-activator of transcription (TAT) protein has been demonstrated for efficient delivery across the BBB^12^,^13^,^14^. Although non-toxic and safe, TAT-mediated delivery technically fails to achieve controlled on-demand delivery across BBB. Among current emerging methods, smart magnetic nanoparticles based approaches such as magnetic-liposome and layer-by-layer formulations are top choices to efficiently deliver therapeutics across the BBB^6^,^7^,^8^,^9^,^17^,^18^,^19^. However, as of now, the delivery of such NFs across the BBB is an existing challenge. Considerable research is required to develop a method to efficiently deliver effective therapeutic NFs to CNS and also retain their efficacy for the treatment of CNS diseases. Therefore, there is a critical requirement to select an efficient and biocompatible nanocarrier, which can be externally guided for on-demand delivery of cargo across the BBB^7^.

In this manuscript, we present for the first time a non-invasive, magnetically guided CNS delivery of magneto-electro carriers (MENCs) of BaTiO_3_@CoF_2_O_4_ (BTO@CFO), 20–30 nm in size, in the brain. We previously explored the delivery of MENCs for on-demand controlled release of anti-HIV drugs as potential therapy against NeuroAIDS using an *in-vitro* model^20^,^21^. Based on cytotoxicity assay performed using MENC doses ranging from 5 to 20 mg/kg, an optimized nontoxic MENCs concentration of 10 mg/kg was injected into adult C57B1/J mice. To ensure CNS delivery, MENCs injection was performed under the environment of a static magnetic field (0.8 T) for 3 hours. Results of the transmission electron microscopy (TEM) showed that MENCs were uniformly distributed in all cell population with minimal agglomeration. Further, organ specific (brain, kidney, liver, lung and spleen) and peripheral blood (Hepatic and renal function test) toxicity were analyzed using standard Hematoxylin and Eosin (H&E) staining method and blood profiling method, respectively and showed no toxicity. Behavioral studies in MENCs injected mice were conducted to evaluate the effect of MENCs in neurocognition using specific neurobehavioral tests. Results of these studies showed no significant impairment in motor coordination when compared to untreated or PBS treated mice. Overall, our findings confirm that MENCs can be delivered across the BBB and exhibit no apparent cytotoxicity or behavioral impairment in the mice, thus suggesting a potential application of MENCs for site-specific, on-demand and controlled delivery of therapeutics across the BBB to treat CNS diseases.

To evaluate the magnetism of the MENCs, magnetic hysteresis loops of the CoFe_2_O_4_ (CFO) and BTO@CFO at room temperature are presented in Fig. 1A. The obtained lower magnetization of MENCs (34 emu/g) compared to CFO (53 emu/g) was due to the deposit of BaTiO_3_ (BTO) onto CFO surfaces and confirmed fabrication of MENCs. MENCs demonstrate ferromagnetic behavior that can be correlated with ferromagnetism and multiferroic, polarization properties of an electro-active material. This is a necessary phenomenon in order to achieve on-demand release through a.c. magnetic field stimulation *via* electromagnetic coils^20^. The particle size of the MENCs was estimated within 20 to 30 nm using TEM analysis (Supplementary Fig. 1). TEM results also confirmed that MENCs are composed of BTO and CFO (Fig. 1B) which is attributed to the appearance of atomic planes related to BTO and CFO (Fig. 1B,a–c). The atomic interplanar spacing of CFO and BTO was estimated using Digital Micrograph Software. The phase purity and crystalline characteristics of MENCs were evaluated using X-ray diffraction (XRD) (Fig. 1C). Observed peaks were indexed and attributed to both CFO (JCPDS 00-022-1086) and BTO (JCPDS 04-001-7269). Obtained diffraction peaks were broad due to the small size of MENCs. XRD pattern confirms that MENC synthesis resulted in CFO and BTO as shown in the crystallographic planes. However, widths and intensities of the peaks are higher at some angles due to the overlapping of CFO and BTO planes. The chemical composition of MENCs, BTO@CFO, was characterized using Raman spectroscopy (Fig. 1D). Among several optically active Raman modes associated with BTO and CFO, the most intense modes characteristic to BTO and CFO were observed, respectively, around 500 cm^−1^ due to dominant tetragonal phase, and 670 cm^−1^ due to cubical inverse-spinel structure^22^. BTO has 12 Raman modes as compared to CFO (5 modes), and therefore BTO dominates the Raman spectra. Due to the shift of Ti ions with respect to oxygen in BTO, the F_1u_ mode splits into A_1_ and E modes, which are further separated into transversal (TO) and longitudinal (LO) components. BTO dominant peaks are observed at 300 cm^−1^ due to E (TO + LO) mixed mode and peak at 515 cm^−1^ due to E (TO) and A_1_ (TO) transversal modes. The peak around 800 cm^−1^ indicates stacking-fault density of BTO, which could be due to the high temperature (780 °C) used during MENCs calcination. Five Raman active modes of CFO, one of A_1g_, one of E_g_ and three of F_2g_ symmetries, are characteristic of the cubic inverse-spinel structure and in agreement with previous reports^23^. The F_2g_ symmetry, characterized by large oxygen motion and very small cobalt displacement, is observed around 670 cm^−1^. The polydispersity index (PDI) value of MENC was estimated as 0.22 ± 0.03 using dynamic light scattering (DLS) method, suggesting that MENCs have good mono-dispersity in PBS. However, the hydrodynamic size of MENC was estimated as 90 nm. Usually the DLS size is higher than TEM size, as nanoparticles were measured in aqueous and dry forms, respectively. The particle size estimated using DLS was higher than the estimate by TEM and XRD due to the hydrophilic nature of MENCs. The Zeta potentials of MENCs in PBS were estimated as −30 mV, suggesting a cationic surface of MENCs.

Our proposed MENCs concentration ranging from 5 to 20 mg/kg was initially used to detect cytotoxicity using primary human astrocytes and SKMNC neuronal cell line. Results of MTT showed that treatments with different concentrations of MENCs exhibited high percentage of viability, more than 90% (for MENCs dose ranging from 0.05 to 0.25 mg/mL) in both cell lines, similar to the untreated control (Fig. 1E,F). However, the MENCs dose of more than 0.25 mg/mL showed lower cell viability ~70%. The MENCs (5 to 15 mg/kg with respect to average mice weight 20 ± 1) do not exhibit any cytotoxicity and are safe for *in-vivo* experiments. 10 mg/kg corresponding to 0.25 mg/mL (10 mg/kg) of MENCs exhibited a maximum of 96% cell viability for both astrocytes and neuronal cells and was selected for injection in C57Bl/J mice (Fig. 1G). An intermediate dose of 10 mg/kg was selected for *in-vivo* application due to easy detection and injection. Lower doses were difficult to detect and higher doses caused difficulty in injection due to high particle-particle interaction.

A qualitative uptake study using *in-situ* TEM imaging (Fig. 2A,B) confirmed that MENCs were capable of efficiently crossing the BBB (Fig. 2A,a control vs Fig. 2B,a). Undoubtedly, abundant amounts of MENCs were found localized into brain cells, including neurons (Fig. 2A,a–c), astrocytes, and microglia. MENCs were also taken up by blood cells (Fig. 2B,a) and smooth muscle cells (Fig. 2B,e). Unlike TAT-mediated delivery across BBB, which resulted in endosomal entrapment of nanocarriers and failure to reach cell nuclei^9^, MENCs were found uniformly distributed inside cells and they were able to reach the nucleus in high numbers. Although navigation of nanoparticles of the nuclei of neuron is very uncommon, Bhardwaj’s *et al*.^24^ recently investigated three of the most commonly used strategies to deliver metal nanoparticles into the nucleus. These findings have been used towards the development of the first intracellular immunosensor using silver nanoparticles based surface-enhanced Raman spectroscopy to detect DNA repair protein. We observed that magnetically-guided delivery showed increased intracellular and nucleus uptake potential than electroporation and cell permeability peptide-facilitated strategies. This approach is a promising non-invasive physical method for high throughput delivery of MENCs and other magnetic particles. Among current competing physical strategies electroporation can be considered somewhat similar to the magnetically-guided delivery, since both use electromagnetic forces. However, our novel method does not require creating any pores to mediate delivery. Consequently, there are little to no chances of cellular damage, which is typical in electroporation of metal nanoparticles as heat generation is a concern^24^.

The ultrathin sections of mice brain, as used for *in-situ* TEM study, were subjected to scanning transmission electron microscopy (STEM) to evaluate elemental and structural analysis of the MENCs localized in the brain (Fig. 2C,D). STEM-based diffraction pattern was recorded at many locations (Fig. 2C,a–f) to explore crystalline integrity of MENCs in mice brain tissues (Fig. 2C,g). STEM images (Fig. 2C,a–f) corresponding to *in-situ* TEM (Fig. 2B,a–f) further confirms the presence of MENCs in BBB (Fig. 2C,a corresponds to Fig. 2B,a), astrocytes (Fig. 2C,b corresponds to Fig. 2B,b), schwann cells surrounding axon terminal (Fig. 2C,c corresponds to Fig. 2B,c), periphery of neuron cells nucleus (Fig. 2C,d corresponds to Fig. 2B,b), microglial cells (Fig. 2C,e corresponds to Fig. 2B,b), and cytosol of astrocytes (Fig. 2.E,f corresponds to Fig. 2B,b). Figure 2C,g shows the convergent beam electron diffraction (CBED) pattern of the MENCs in the brain cells in STEM mode to explore structural integrity. CBED pattern was indexed based on CFO (JCPDS 00-022-1086) and BTO (JCPDS 04-001-7269), and results confirmed the presence of MENCs inside the brain cells.

Brain tissues were also scanned at various positions and an identical energy-dispersive spectroscopy (EDS) spectrum was obtained for elemental analysis of the MENC. STEM images (Fig. 2D,a–c) depict MENC presence in arterioles and smooth muscle cells. A single spot from each image was selected for EDS analysis (Fig. 2D,d). The elemental analysis showed the presence of Ba, Ti, Co and Fe ions confirming that MENC does not lose its local chemical environment during transmigration across the intricate BBB. EDS was conducted in both TEM and STEM modes. The results were identical and indicated the presence of the Os, which was used for fixation and staining of mice tissue for *in-situ* TEM study, Ni from the TEM grid, C, O, Cl primarily from the organic matter of the cell, and Co, Fe, Ba, and Ti from both CoFe_2_O_4_ (CFO) and BaTiO_3_ (BTO). The obtained relatively very low intensity of Ba and Ti was due to merely a very thin layer/shell of BTO surrounding CFO core. The results of CBED and EDS confirmed that MENC does not disintegrate during the complex process of CNS delivery.

A quantitative estimation of MENCs across BBB was performed using inductively coupled plasma mass spectroscopy (ICP-MS). Known concentrations of Ti (0, 10, 25, 50, 60, 80 and 100 ppb or μg/L) and Fe (0, 10, 25, 50, 60, 80 and 100 ppb or μg/L) in liquid suspension were used to establish calibration curves using ICP-MS (Supplementary Fig. 2A,B). We selected Ti (wrt BTO) and Fe (wrt CFO) to confirm that MENCs do not loose chemical structure during CNS navigation. A good linearity was obtained for both elements with a regression coefficient (r^2^) of ~0.998. ICP-MS study was conducted using a known concentration of MENCs to estimate the percentage of Fe content using a related calibration curve. Results confirmed that MENCs consist of 36.5% Fe content. Established calibration curves were also used to estimate the concentration of MENCs reaching mice brains. The brains of control mice were processed for ICP-MS study using identical conditions. Results of the ICP-MS study confirmed that the weights of Ti in control and injected mice brains were 141 and 149 μg/g tissue, respectively, and the weights of Fe in control and injected mice brains were 623 and 683 μg/g tissue, respectively. Results confirmed that concentrations of MENCs in mice brains was 38 μg/g tissue.

Histopathology analysis (Fig. 3A) showed that MENCs injected mice undergo minor extramedullary hematopoiesis as compared to control mice. In addition, H&E staining did not demonstrate any recruitment of macrophages or other immune cells in liver (Fig. 3A,a), kidney (Fig. 3A,b), spleen (Fig. 3A,c), and brain (Fig. 3A,d), indicating lack of toxicity. Results also confirmed that major organs of MENCs injected mice did not have any abnormality at cellular and organ levels. Moreover, the H&E staining of control and MENP-injected mice lung tissue (Supplementary Fig. 3) did not exhibit toxicity similar to brain, liver, spleen and kidney. This indicates no dramatic change happening during the process. Specific attention was given to the enzyme levels in order to best assess any evidence for induced toxicities. Blood biochemical tests (hemoglobin, creatinine, electrolytes, sodium, potassium, chloride, alanine aminotransferase, alkaline phosphatase, total serum calcium and phosphate) and liver function tests (albumin, transaminases [alanine, aspartate and total bilirubin], gamma-glutamyl transferase and alkaline phosphatase) were performed; all values were found within normal reference range (Fig. 3B). Overall the outcomes of above studies confirm that the delivery of MENCs is safe for both *in-vitro* and *in-vivo* systems.

Based on the distribution of nanoparticles in different mouse brain regions, sensorimotor coordination was measured in mice injected with PBS, MENCs and no injection as the control group, using grip strength, horizontal bar and accelerating rotarod (Deacon) after days 2 and 7 post-treatment. As shown in Fig. 3C,a, mice injected with MENCs (10 mg/kg) showed no difference, within the group, in forelimb grip strength after day 2 and day 7 of post-injection when compared to PBS and no injection control groups. Forelimb grip strength was further tested using two horizontal bars and the combined scores from the 2 and 4 mm bars showed no apparent differences in test performances within groups or between the three groups at day 2 and day 7 (Fig. 3B,b). Furthermore, no differences in motor coordination were evident between the different treated animals, or when MENCs injected animals were compared to fall latency of mice injected with PBS and no injection controls at days 2 and 7 (Fig. 3C,c). Overall, presented results showed no impairment in motor performance between animals that were injected with MENCs, PBS or not injected control groups. The grip strength and rotarod test experiments using C57B1/J mice are demonstrated in the videos (Supplementary video A–C).

In conclusion, our method has demonstrated for the first time the navigation of crystalline, non-toxic and ferromagnetic MENCs, as a potential drug carrier and a.c. magnetic stimuli-responsive for on-demand release feature across the BBB in mice. Results of our study confirmed that MENCs are distributed uniformly in mice brain, with a high uptake and trafficking at the nuclei level of cells. Administration of MENCs exhibited no toxicity in five major organs and blood profile studies. Our study, confirmed that MENCs are capable of crossing the tight junctions of the BBB, and are safe for CNS cells. Additionally, the chemical integrity and structure of MENCs were not compromised during this complex navigation process, showing that the efficacy of nanocarriers was retained. Furthermore, administration of MENCs does not cause any impairment in sensorimotor performance in mice even after 7 days post-treatment in comparison with the control. The presented BBB delivery method is non-invasive and completely safe for *in vivo* application and can therefore be applied for translational research. Herein, the explored salient features support MENCs to be used as potential nanocarriers to deliver therapeutic agents across the BBB to treat CNS diseases such as Alzheimer’s, brain tumors, and NeuroAIDS.

## Methods

### Synthesis and characterization of MENCs

MENCs (BaTiO_3_@CoFe_2_O_4_) synthesis is a 3 step process as described in our previous publications^20^,^21^. In brief, CoFe_2_O_4_ nanoparticles were prepared using a hydrothermal method. Step 1), 15 mL of aqueous mixture of 0.058 g of Co(NO_3_)_2_.6H_2_0 + 0.16 g of Fe(NO_3_)_3_.9H_2_0 was combined with a second mixture of 0.2 g of polyvinylpyrrolidone (Avg. mol wt−40,000) dissolved in a 5 mL of aqueous solution with 0.9 g of sodium borohydride and heated at 120 °C for 12 h. Step 2), the precursor solution of BaTiO_3_ was prepared by mixing a 30 mL aqueous solution containing 0.029 g of BaCO_3_ and 0.1 g of citric acid with a 30 mL ethanol solution containing 0.048 mL titanium isopropoxide and 1 g of citric acid. Steps 3), the BaTiO_3_@CoFe_2_O_4_ MENCs were prepared by dispersing 0.1 g of CoFe_2_O_4_ nanoparticles in the precursor solution obtained in step 2. The suspension of both counterpart nanoparticles were sonicated for 2 h. The well-dispersed mixture was dried at 60 °C overnight. Dried MENCs were allowed to calcine at 780 °C for 5 h. The average diameter of MENCs was controlled to be between 20 to 30 nm.

The particle size, distribution, morphology, and crystallinity of MENCs were studied using an FEI CM 200 transmission electron microscope. The phase composition of synthesized MENCs was studied using an X-ray Diffractometer (based on Mo-Kα radiation). Diffraction patterns of MENCs were analyzed and indexed using ICDD PDF 2014 database and Match software. The chemical fingerprint of the MENCs was studied using Raman Spectro-microscope (Nomadic Raman microscope with BaySpec 532 nm laser). 20 μL of a 10 mg/mL aqueous solution of MENCs were drop casted on silica substrate to acquire Raman spectra.

### Cytotoxicity study *(in-vitro)*

**Figure 1:**
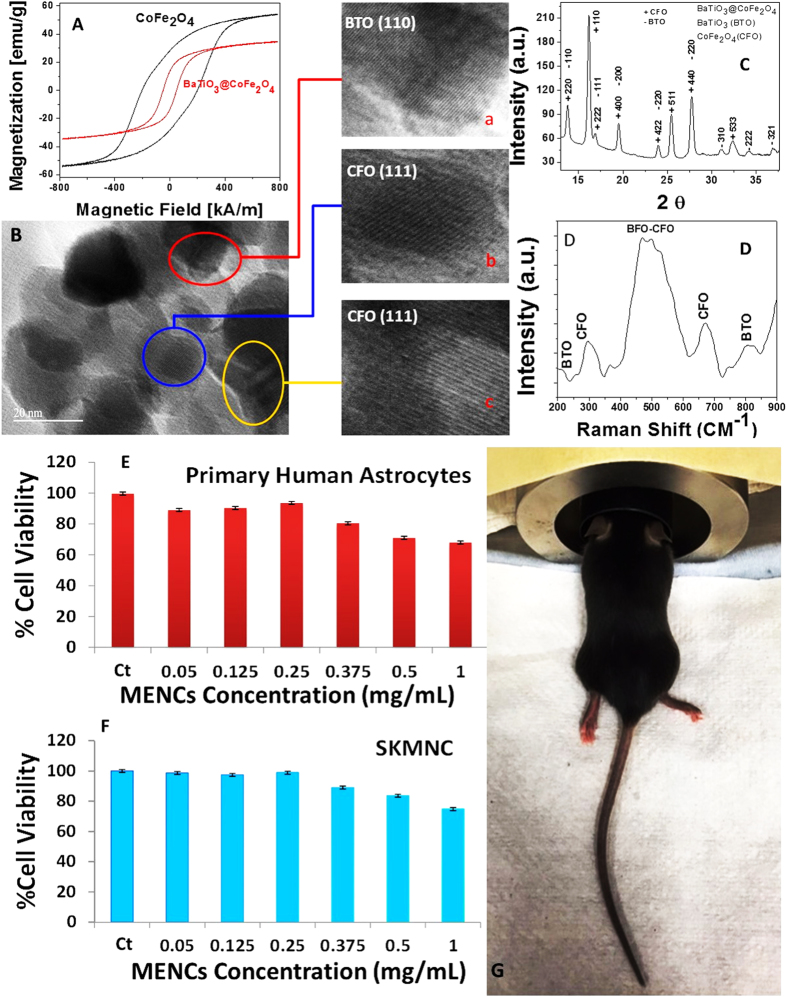
Characterization and *in-vitro* toxicity of MENCs. (**A**) VSM study of CoFe_2_O_4_ and MENCs (BaTiO_3_@CoFe_2_O_4_). (**B**) TEM image of synthesized MENCs, atomic planes with respect to BTO (a) and CFO (b–c) reveals formation of MENCs. (**C**) X-ray diffraction pattern of MENCs to explain phase purity and crystallinity of MENCs, (**D**) Raman spectra of MENCs to explain the functionality of MENCs (h), Our proposed doses (2.5, 5, 10, 15 20, 20 mg/kg back calculated as 0.05, 0.125, 0.25, 0.375, 0.5, and 1 mg/mL respectively with respect to average mice weight 20 ± 1) approved by IACUC were selected for *in-vitro* toxicity evaluation of MENCs using MTT assay based on primary human astrocytes (**E**) and SKMNC (**F**), significance was considered to be p < 0.05. Demonstration of mice injection bed (**G**). An optimized dose of 10 mg/kg with respect to 20 ± 5 g of mice, corresponds to 0.25 mg/mL was injected in mice. For MENCs injection, mice was under anesthesia and after injection the physical condition of mice was under continuous monitoring.

An MTT [3-(4,5-Dimethylthiazol-2-yl)-2,5-Diphenyltetrazolium Bromide] assay was used to study *in-vitro* cytotoxicity. Human astrocytes and SKNMC (1 × 10^6^ cells/well) were grown in 6-well plates. Grown cells were treated with 100 μL of various MENCs doses (0.05–1 mg/mL). We consider IACUC approved doses (5 to 20 mg/kg) for this research. For MTT assay, MENCs doses were back calculated with respect to the average mice weight of ~20 ± 1 gm as shown in Fig. 1E,F. Moreover, one dose less than 5 mg/kg and one higher than 20 mg/kg were also considered for MTT assay for better understanding.

Further, well plates were maintained in a humidifier incubator with an internal environmental consisting in 95% air and 5% CO_2_ at 37 °C. After 48 days of incubation, one mL medium supplemented with 100 μL of MTT (100 mg MTT/20 mL PBS) was added to each well and incubated at 37 °C for 3 hrs. Later, one volume of detergent reagent (20% SDS in 50% DMF) was added, rocked for about 2 h, and then centrifuged. The optical density of the solubilized formazan was determined using UV-Vis spectrophotometer by measuring absorbance at 550 nm. The optical density of formazan in each well is directly proportional to the cell viability, utilized for calculations.

### Cytotoxicity study of MENCs (*in-vivo*)

All experiments were approved by the Institutional Animal Care and Use Committee at Florida International University in accordance with the National Institutes of Health’s Guide for the Care and Use of Laboratory Animals. An optimized dose of MENCs (10 mg/kg with respect to 20 ± 1 g mice,) was used for injection in C57B1/J mice (n = 6 /group, control n = 6/group, males and 6 weeks old) in all experiments. (Charles River Laboratory, Inc., Wilmington, MA). MENCs were suspended in phosphate-buffered saline (PBS) in order to make an injection suspension. A single dose (10 mg/kg) of MENCs was administered intravenously (i.v.-administration) in each mice and placed their head in stable external magnetic field (0.8 T) under sedated condition for 3 h (Fig. 1G). The injected dose was selected to correspond to a human dose of 6.5−20.3 mg/kg by interspecies allometric scaling factor. After 3 hrs of incubation, mice were kept at the normal cage condition under observation for a week. Intermittent blood samples were collected at days 2 and 7 in order to check Hematoxylin and eosin (H&E) staining and blood toxicity. The plasma supernatant was stored at −80 °C for analysis. Serum samples were analyzed for liver and renal panel toxicity from VRL, Maryland. After blood collection, mice were harvested to collect major organs such as brain, liver, kidneys, and spleen for histopathology. Histopathology analysis was done with H&E staining to observe any systemic toxicity in these tissues at University of Miami tissue core facility.

### *In-vivo* TEM studies of brain tissue

FEI CM 200 TEM was used for the morphological characterization of brain tissue of control and MENCs injected brain tissue samples to evaluate MENCs transmigration, particles size distribution inside brain and its uptake within the CNS cells. An *In-vivo* TEM study was also performed on PBS injected mice brain tissue using identical experimental conditions. Following the animal perfusion protocol^25^, the mouse skull was chipped-off and tweezers were used to remove the brain and each of the two hemispheres was cut into 8 transverse blocks. The blocks were processed and analyzed for qualitative and quantitative uptake of MENCs using *in-situ* TEM and ICPMS, respectively. Protocols used to process brain tissue samples for these studies were adapted from a previous report^24^.

For *in-situ* TEM experiments, brain tissue blocks were cut into smaller sizes (~50 μm thick), rinsed in ice-cold PBS three times, and fixed using 2% gluteraldehyde (primary fixative for 90 minutes) and 1% Osmium tetraoxide (secondary fixative for 30 minutes) with washing in between each individual fixation. Samples were transferred into watch glasses for serial dehydration, 35, 50, 70, 80, 90, 95 and 100%, using histology grade absolute alcohol for 20–30 minutes each. Dehydrated samples were embedded into Spurr’s epoxy resin following manufacturer’s guidelines, mixing components ERL, DER, NSA and DMAE in ratio 30:23:80:1. Samples were infiltrated using a series of resin: ethanol dilutions, 1:2, 1:1 and 3:1 and 100% resin for 3–5 hrs at each step. Sections were transferred into molds, filled with resin, and allowed to polymerize overnight at 70 °C in an Enviro-Genei incubator to obtain an isosceles trapezoid shape. Blocks were trimmed using a blade and cut into ultrathin sections (≤50 nm) using an ultra microtome (Porter-Blum MT-1, DuPont-Sorvall, USA) and a diamond knife (DDK, USA). Sections were collected in boats filled with acetone to help section stretching (ribbon-like) and were loaded on Ni grids with handles to allow ease of handling and robustness. The grids with samples were allowed to dry, observed under a light microscope to select the best samples, and stored in a grid box until TEM analysis.

### Inductively coupled plasma mass spectroscopy of mice brain tissue

The ICP-MS studies were performed using Perkin Elmer Sciex, model ELAN DRC-II at the FIU Trace Elemental Analysis Facility. ICP RF power 1375, nebulizer gas flow 0.90 L/min, plasma gas flow 16 L/ min, and lens voltage 8.25 V were selected as the acquisition parameter for each measurement. ICP-MS studies were performed to estimate Fe ion content in MENCs and to estimate MENCs concentration in MENCs injected mice brain.

### Estimation of Fe ion concentration in MENC

To establish references for estimating ion concentrations of MENCs, calibration curves with respect to Ti and Fe ion were founded. MENCs suspended in PBS media were considered a blank negative control. Prior to ICP-MS analysis, samples were dissolved using the following acid digestion protocol: **1)** Vortex the suspension and immediately place 20 μL of the suspended nanoparticles into a digestion polypropylene tube, and add 1000 μL of 16 M nitric acid (70%, optima grade), **2)** Heat the samples in a dry heater block at 90 °C for 1 hour. Cover the vials with the digestion cap to avoid loss of volatile compounds. Check the samples to avoid complete dryness, **3)** Remove the samples from the heater block and let them cool down at room temperature for several minutes, **4)** Add 250 μL of hydrogen peroxide (30%, optima grade) and heat at 90 °C for 30 minutes, **5)** Remove the samples from the heater block and let them cool down at room temperature for several minutes, **6)** If needed, add 250 μL of hydrogen peroxide (30%, optima grade) and heat at 90 °C for 30 min until dry, **7**) Remove the samples from the heater block and cool at room temperature, **8)** Add 25 μL of Sc 10 ppm and dilute samples with nitric acid 0.8 M to a final volume of 10 mL, followed by sonication for 15 minutes. Close the vial caps and vortex. This first dilution (1:500) is used to measure the content of Ti in the sample, **9)** for analysis of Fe, a total dilution factor of 1:10000 is required. Reconstitute the dry sample to 10 mL with nitric acid 0.8 M, then take an aliquot of 500 μL, add 25 μL of Sc 10 ppm and dilute to 10 mL. All samples were spiked with the internal standard (Sc). Each sample was analyzed in 6 to 9 replicates. Concentrations of Fe and Ti ions were in liquid suspension. A known concentration of MENCs (2 mg/mL) was used for ICP-MS study to measure Fe content in the formulation of MENPs.

### Estimation of MENCs concentration in mice brain

Control and MENCs injected mice brain tissues were sliced and kept in a 1% formaldehyde solution. To remove water content, all samples were processed via serial dehydration using 24, 40, 60, 80, 100% ethanol. Prior to ICP-MS analysis the samples were digested using identical acid digestion process as used for MENCs Previously established calibration curves for Ti and Fe were used to estimate ion concentration in control and MENCs injected mice brain tissues.

For both experiments, optima grade nitric acid was used to prepare the calibration curve and standards. Three reagent blanks were exposed to the same digestion and dilution process as the rest of the samples. Quality control standards were prepared at concentrations of 25 and 50 ppb and analyzed at the beginning, middle and end of the analytical sequence. All quality control checks passed our quality control criteria (precision and bias better than 10%). Instrument blanks and reagent blanks were analyzed and the concentration values for the samples were then reported after their respective background subtraction.

### Validation of MENCs presence in brain using STEM and CBED

In order to evaluate the chemical analysis of injected MENCs and their distribution inside the brain, FEI Tecnai F30 high-resolution transmission electron microscope (HRTEM) was employed in both TEM and STEM (Scanning Transmission Electron Microscopy) modes using the energy dispersive spectroscopy (EDS) technique. Selected area electron diffraction (SAED) and convergent beam electron diffraction (CBED) patterns were obtained in TEM and STEM modes, respectively, to confirm the presence of the MENCs in the brain cells. The sampling for STEM study was similarly adapted to TEM studies.

### Histopathology of Mice

Tissue samples of brain, liver, kidney and spleen were fixed in 4% phosphate-buffered formaldehyde and paraffin-embedded according to conventional methods. For histopathological analysis, tissue sections were stained with hematoxylin and eosin (H&E). All systemic tissue toxicity was performed at the University of Miami’s Department of Pathology. Histopathological evaluations were performed in accordance with the guidelines of the Society of Toxicologic Pathology. Images were taken using a Carl Zeiss Axio. All microscopic images were captured using an AxioCam MRc5 CCD camera.

### Blood toxicity profile

For blood toxicity analysis (n = 3 for both group of experiments), blood was collected using cardiac puncture at days 2 and 7 after the initial treatment. Serum chemistry profiles were analyzed through VRL, Maryland. The changes in enzyme levels were analyzed based on the normal range provided by VRL.

### Neurobehavioral Study of MENs injected mice

Male C57Bl/J mice of 6 weeks (n = 6 for each experiment and each group) were purchased from Jackson Laboratory and housed in standard ventilated cages with free access to water and food in a climate-controlled environment on a 12-h light⁄dark cycle (lights off at 09:00 h). Before testing, body weight was measured and each mice was habituated to the environment for 20–30 minutes. Motor performance was evaluated using a combination of grip strength, horizontal bar and rotarod tests as described by Deacon 2013. A) Grip strength allowed us to evaluate for motor weakness as this test relies on the instinctive tendency of the mouse to grasp an object with its forelimb. Grip strength was determined by placing each mouse with 2 limbs on a grid attached to a force gauge and steadily pulling the mouse by its tail. The grip force was automatically recorded using a computerized grip strength meter (SDI Grip Strength; San Diego Instruments, San Diego, CA, USA). For each measurement, the test was performed in triplicate at 1 minute intervals, and an average of the values were taken and used as the animal’s measure of grip strength. B) Forelimb strength and coordination were further evaluated using 2 and 4 mm diameter horizontal bars. Mice were tested for their ability to hold onto the bar and/or touch the ends of the pole (supporting the bar). Falling after 30 seconds or placing one forepaw on a bar support without falling gave the mouse the highest score of 5. After the mice were scored for the 2 mm bar, it was then tested on the 4 mm bar, and the scores on the two bars were added. C) An accelerated rotating rod test allowed us to evaluate coordination and motor skill acquisition (RotaRod-5; San Diego Instruments, San Diego, CA, USA). Mice were placed on the rod without any training period and the rod was accelerated from 1 to 40 rpm in 1.0 rpm steps per 15 s. The time the mice spent on the rod without falling was recorded. All animal studies were approved by the Institutional Animal Care and Use Committee (IACUC) at Florida International University.

## Additional Information

**How to cite this article**: Kaushik, A. *et al*. Magnetically guided central nervous system delivery and toxicity evaluation of magneto-electric nanocarriers. *Sci. Rep*. **6**, 25309; doi: 10.1038/srep25309 (2016).

## Supplementary Material

Supplementary Information

Supplementary Video A

Supplementary Video B

Supplementary Video C

## Figures and Tables

**Figure 2:**
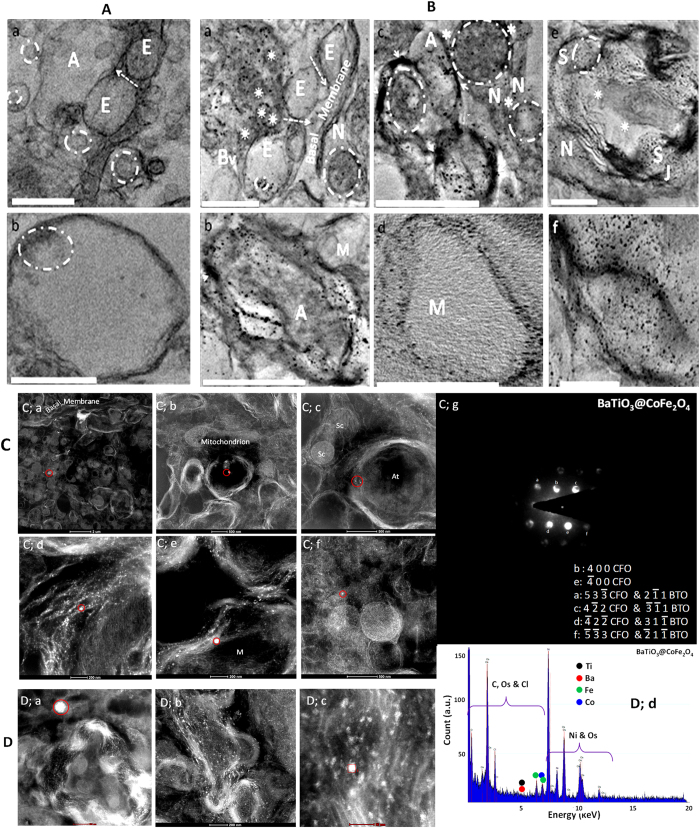
MENCs navigation across BBB (**A**,**B**), and their elemental analysis (**C**,**D**) *in situ* by TEM, STEM and CBED. (**A,B)** *In situ* TEM image of mouse’s brain tissue without MENCs injection (A, control) and after MENCs injection (B, treatment). MENCs are capable of navigating across BBB (A,a vs B,a), direction of movement across tight junctions of endothelial (**E**) cells layer is indicated by arrows. MENCs are able to reach target sites, including neurons (**N**), astrocytes (**A**) and microglia (**M**), and are also observed in smooth muscle cells (**S**), endothelial cells (**E**) and blood cells (☼). Most MENCs are uniformly distributed in brain tissue/cells and are able to reach nucleus (dotted circles), but some agglomeration of MENCs in cell membranes and their entrapment in endosomes is also observed (solid arrow heads). * represent synapses (**B**,c), J represents neuromuscular junction between S and at (axon terminal, B,e), A layer of schwann cells (sc) surrounding at is also observed (**B**,c). Scale bars: 1 μm (A,a; B,a,b,e) and 0.5 μm (A,b; B,c,d,f). (**C,D**) Validation of MENCs chemical integrity and stability inside brain: *In situ* STEM confirms elemental analysis and distribution of MENCs inside brain (**C**,**D**). Various spots ware selected for both CBED and EDS measurement. Each spot, highlighted by red circle, was analyzed morphologically to understand MENCs distribution in brain cells. STEM-based CBED pattern (obtained from C;a–f) were analyzed for the evaluation of MENCs crystallinity at brain (C;g), the zone axis is [011] direction, and EDS spectra (obtained from D;a–c) for elemental analysis of MENCs in brain tissue samples (D;d).

**Figure 3:**
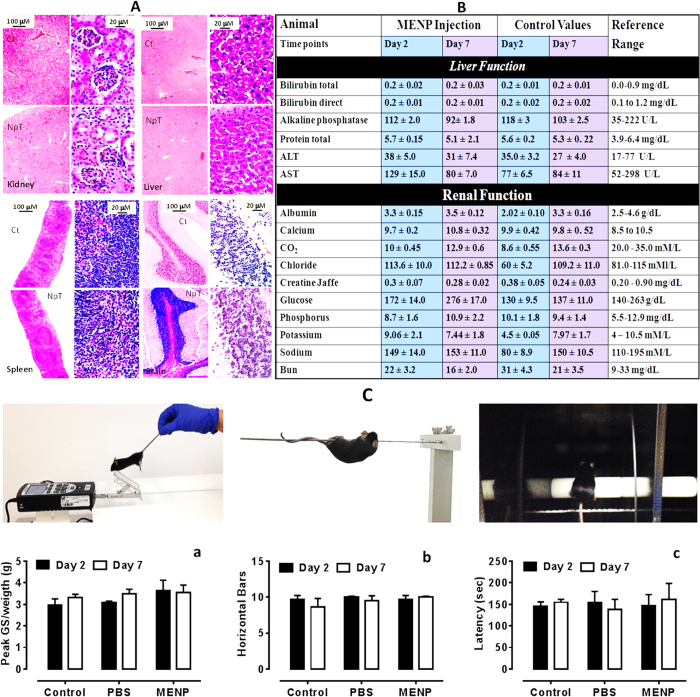
Toxicity and neurobehavioral evaluation of MENCs injected mice. (**A)** Histopathology of PBS injected (as control i.e., Ct) and MENCs injected mice (NpT) with respect to brain (n = 3). (**B**) Blood toxicity analysis of MENCs injected mice (NpT) at day 2 and 7 (n = 3). The average value of various parameters of liver and renal function were studies and compared reference cut off. Histopathology and blood toxicity profile showed no-toxicity at 10 mg/kg of MENCs (n = 3). (**C**) Sensorimotor activities after days 2 and 7 of MENCs challenge. Indicated treatments are: Control (no injection), PBS (saline, I.V.) and MENCs (10 mg/kg, I.V.). Mice tested after day 2 were re-tested at day 7 and the results from each day are shown. (**a**) Grip strength. The average forelimb grip strength of each animal was normalized to its corresponding body weight. (**b**) Horizontal bar. Each score represents the combined duration of a mouse on the 2 mm and 4 mm bars. (**c**) Accelerating rotarod. The time (sec) represents the latency to fall and the time each mouse spent on the rotating rod. All data are presented as the mean ± the standard error of the mean (S.E.M.). The results were analyzed within group comparisons (days 2 and 7) using t-tests and across group (Control, PBS, MENCs) comparisons for each day using one-way analysis of variance (ANOVA) followed by Tukey’s post-hoc tests to determine statistical significance (Graph Pad 5 Software, Inc., La Jolla, CA, USA). Significance was considered to be p < 0.05.
