# Supplementary material for: Magnetically guided central nervous system delivery and toxicity evaluation of magneto-electric nanocarriers
Source: Sci Rep. 2016 May 4;6:25309. doi: 10.1038/srep25309 (PMC4855156; doi:10.1038/srep25309)

Magnetically guided central nervous system delivery and toxicity evaluation of magneto-electric nanocarriers

Ajeet Kaushik[a], Rahul D. Jayant[a], Roozbeh Nikkhah-Moshaie[b], Vinay Bhardwaj[c], Upal Roy[a], Zaohua Huang[a], Ariel Ruiz[a], Adriana Yndart[a], Venkata Atluri[a], Nazira El-Hage[a], Kamel Khalili[d], Madhavan Nair[a]*

[a] Center for Personalized Nanomedicine, Institute of Neuroimmune Pharmacology, Department of Immunology, Herbert Wertheim College of Medicine, Miami, FL-33199, USA

[b] Department of Mechanical & Materials Engineering, Florida International University, Miami, FL-33199, USA

[c] Department of Biomedical Engineering, Florida International University, Miami, FL-33199, USA

[d] Department of Neuroscience, Center for Neurovirology and Comprehensive NeuroAIDS Center, Lewis Katz School of Medicine, Temple University, Philadelphia, PA-19140, USA

---

*Corresponding Author: nairm@fiu.edu (M.N.)

Legends for supplementary figure

Fig S-1: Particles size distribution of MENCs

Fig S-2: ICP-MS study to quantify MENCs concentration at brain. Two calibration curves were established with respect to Ti (A) and Fe (B) ion known concentration. These curves were used estimate concentration of Ti and Fe ion in control and MENCs injected mice.

Fig S-3. H&E staining of control (A) and MENCs injected (B) mice lung.

Video Legends

Video S-A: Demonstration sensorimotor activities of mice after days 2 and 7 of MENCs challenge. Indicated treatments are: Control (no injection), PBS (saline, I.V.) and MENCs (10 mg/kg, I.V.). Mice tested after day 2 were re-tested at day 7 and the results from each day are shown Grip strength,

Video S-B: Demonstration sensorimotor activities of mice after days 2 and 7 of MENCs challenge. Indicated treatments are: Control (no injection), PBS (saline, I.V.) and MENCs (10 mg/kg, I.V.). Mice tested after day 2 were re-tested at day 7 and the results from each day are shown Horizontal bar,

Video S-C: Demonstration sensorimotor activities of mice after days 2 and 7 of MENCs challenge. Indicated treatments are: Control (no injection), PBS (saline, I.V.) and MENCs (10 mg/kg, I.V.). Mice tested after day 2 were re-tested at day 7 and the results from each day are shown Accelerating rotarod.

Fig S-1:

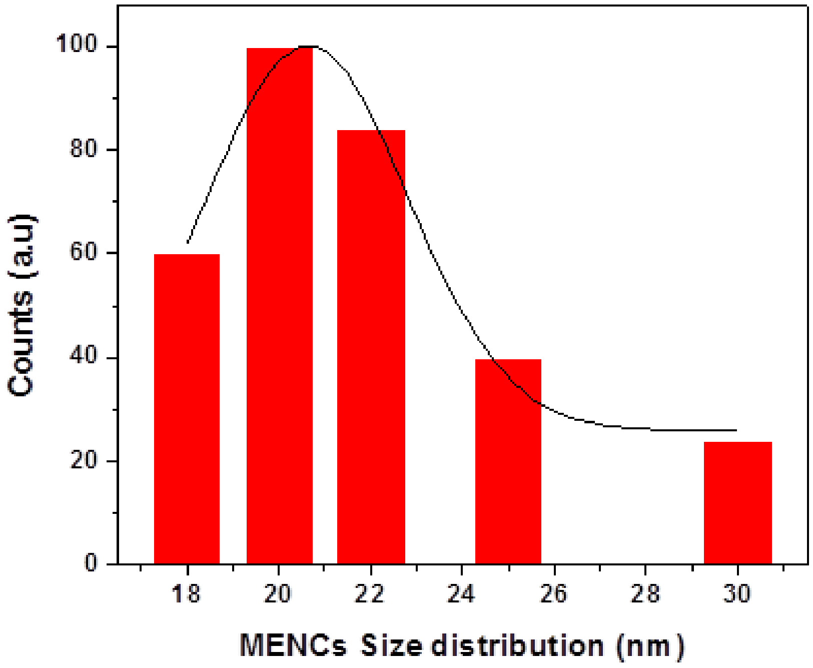

100
80
60
40
20
0
Counts (a.u)
18
20
22
24
26
28
30
MENCs Size distribution (nm)

Fig S-2

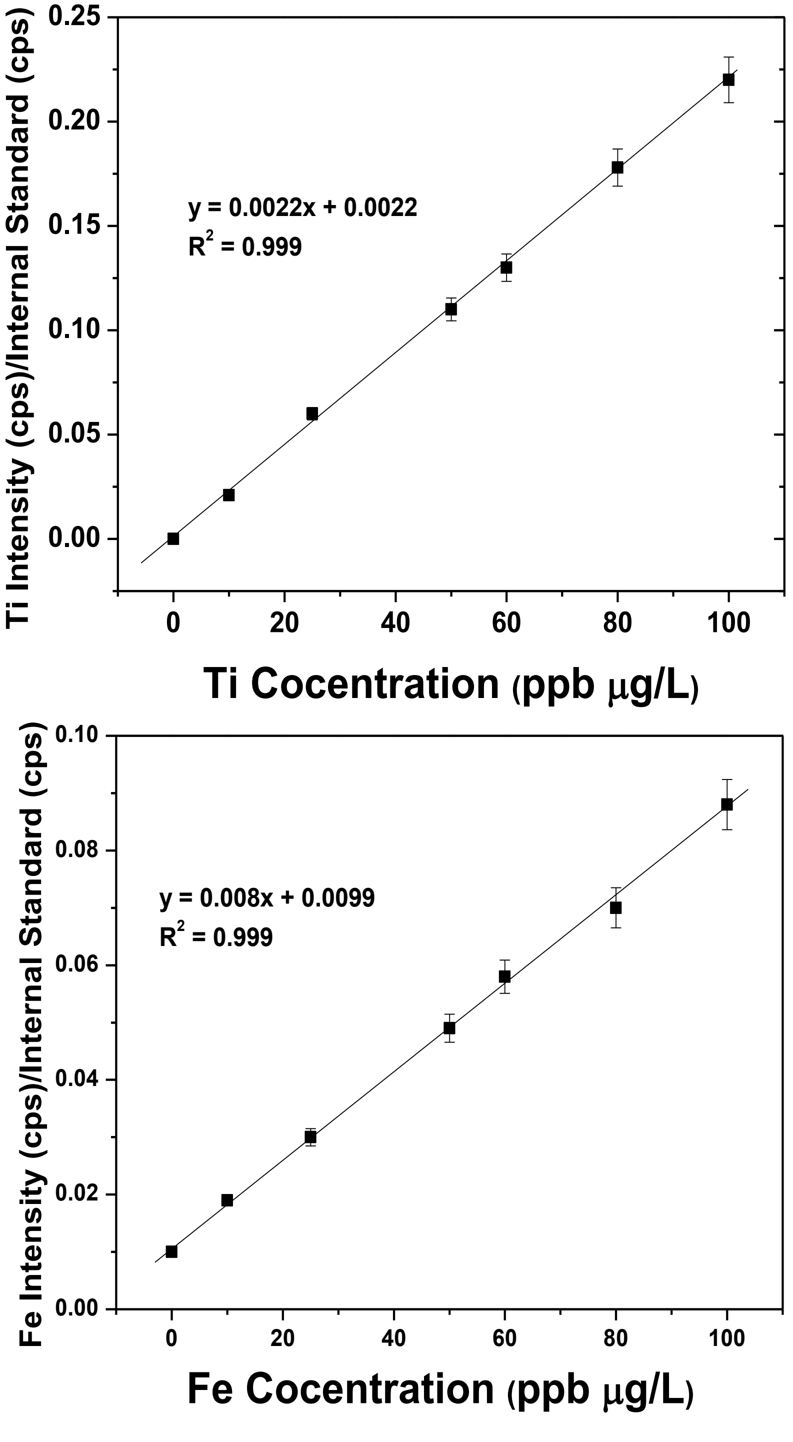

y = 0.0022x + 0.0022
R² = 0.999
Ti Intensity (cps)/Internal Standard (cps)
Ti Cocentration (ppb μg/L)
0.25
0.20
0.15
0.10
0.05
0.00
0
20
40
60
80
100
y = 0.008x + 0.0099
R² = 0.999
Fe Intensity (cps)/Internal Standard (cps)
Fe Cocentration (ppb μg/L)
0.10
0.08
0.06
0.04
0.02
0.00
0
20
40
60
80
100

Fig S-3

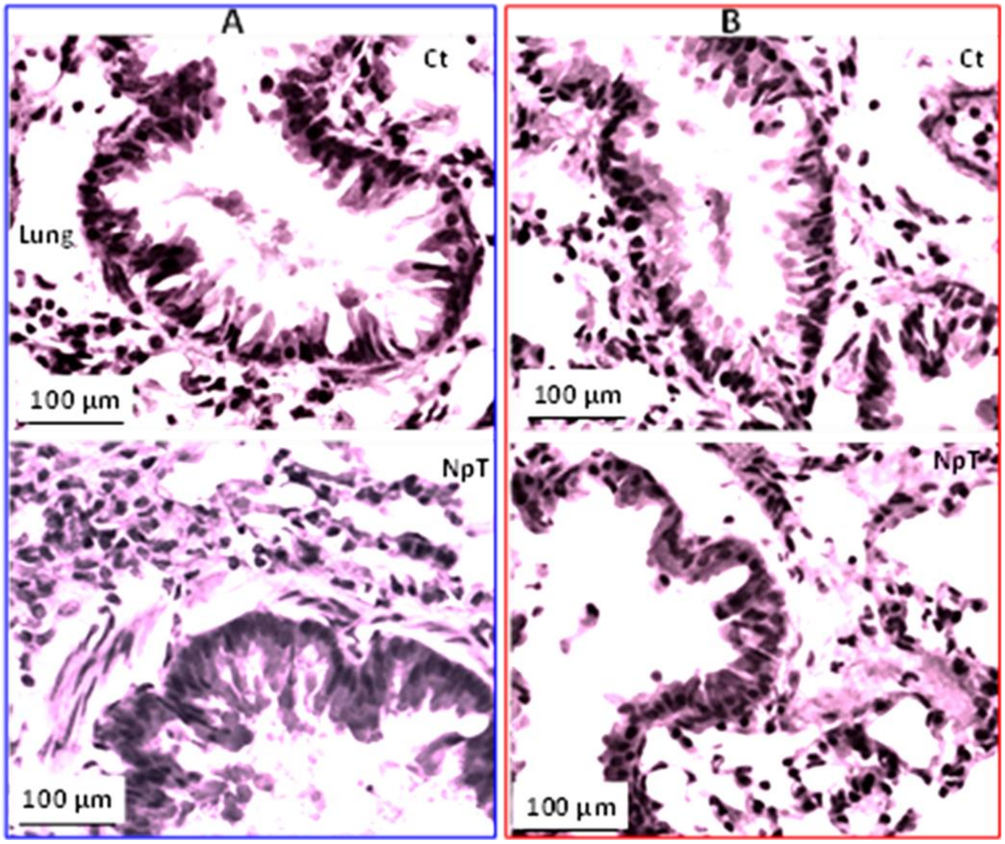

Supplement: Supplementary Information [file srep25309-s1.pdf]
